# Cryotherapy in Knee Osteoarthritis: A Systematic Review With Meta‐Analysis

**DOI:** 10.1111/papr.70055

**Published:** 2025-06-16

**Authors:** Rebeca Ferreira Dias, Sarah de Lima Silva, Sophia Parreira de Mello, Lucinar Jupir Forner Flores, Márcia Rosângela Buzanello, Gladson Ricardo Flor Bertolini

**Affiliations:** ^1^ The State University of Western Paraná (UNIOESTE) Cascavel Brazil

**Keywords:** cryotherapy, knee osteoarthritis, systematic review

## Abstract

**Objective:**

To update knowledge on the effects of cryotherapy in reducing pain, increasing strength, and improving function in patients with knee osteoarthritis.

**Methods:**

The databases used included PubMed, Embase, Cochrane, Physiotherapy Evidence Database (PEDro), Scopus, Web of Science, and LILACS. In addition, gray literature was searched in Google Scholar, LIVIVO, Open Gray, and the CAPES Library of Theses and Dissertations. The risk of bias was assessed using the Cochrane tool, RoB 2, by two independent reviewers, with conflicts being resolved by consensus. The primary outcome was pain, while secondary outcomes included functionality and muscle strength.

**Results:**

Five randomized controlled trials were selected from 2094 initial registrations. The meta‐analysis included all five studies in the pain intensity outcome, resulting in a standardized mean difference (SMD) of −0.57 (95% CI: [−0.97, −0.18]; *p* = 0.004; I^2^ = 42%). In addition, four studies were included in the functionality outcome, with an SMD of −0.28 (95% CI: [−0.58, 0.02]; *p* = 0.07; I^2^ = 0%). Only one study assessed muscle strength.

**Conclusion:**

These findings indicate that cryotherapy can be useful as part of a comprehensive treatment for knee osteoarthritis, especially when combined with kinesiotherapy, but its effectiveness as a stand‐alone treatment still requires further studies with a lower risk of bias.

## 1 Introduction

Osteoarthritis (OA) is a chronic, degenerative condition of the joints, marked by deterioration of the articular cartilage and impairment of the surrounding tissues [1, 2, 3]. The symptoms of this disease are associated with pain and functional limitations, as well as morning stiffness, crepitus, reduced joint and muscle range of motion, reduced muscle trophism and ligament overload [2, 4]. It is the most prevalent progressive musculoskeletal condition, with the highest incidence observed in the hips and knees, and is mainly characterized by structural changes in the articular cartilage and subchondral bone. It has grown significantly in recent decades, reflecting alarming data related to disability, reduced quality of life and the burden on the health system [4, 5, 6].

Knee OA (KOA) continues to expand, partly due to the increased prevalence of obesity and other risk factors, such as: advanced age, previous history of injuries, obesity, joint misalignment and instabilities that result in greater mechanical stress, repetitive activities, professional sports practice and physical inactivity, which is considered a mild risk factor, as it can make knee joints less stable and more susceptible to injury [4]. Treatment is variable and depends on the stage of the disease, with surgical options such as arthroscopy, osteotomies and arthroplasties being the last line of treatment [7]. The initial choice usually falls on conservative forms, with the use of anti‐inflammatory drugs, Tai Chi, Yoga, orthoses and physiotherapeutic resources such as manual therapy, thermotherapy, photobiomodulation, physical exercises, shock waves, in isolated or associated forms, being extremely common [7, 8, 9, 10, 11], and although it's not a first‐choice resource, cryotherapy can be mentioned [12], which is much more common in cases of knee arthroplasty [13, 14, 15].

The aim of cryotherapy is to reduce body temperature, and different means are used for this purpose. With this reduction, it is possible to reduce tissue metabolism, blood flow, and nerve conduction, generating therapeutic effects such as reducing pain stimuli, edema, and inflammatory processes [13, 16, 17, 18]. Although there are systematic reviews that address the use of cryotherapy in individuals with knee osteoarthritis, the resource is addressed among other physical resources [12, 19], or in the research in which it was addressed exclusively, when it was carried out, the authors pointed out that there was not enough data to draw conclusions about its therapeutic efficacy [20]. Therefore, due to possible new articles being added by the distance of the publication time, as well as publications in the gray literature, a new search of available data was conducted in order to update knowledge on the effects of cryotherapy in reducing pain, increasing strength, and improving function in patients with knee osteoarthritis.

## 2 Materials and Methods

### 2.1 Protocol

This review was prepared according to the Preferred Reporting Items for Systematic Reviews and Meta‐Analyses (PRISMA) flowchart. The protocol for this systematic review was registered on OSF https://doi.org/10.17605/OSF.IO/6YM4R.

### 2.2 Eligibility Criteria

The acronym PICOS was used to formulate the question focused on in this study. P (population)—adult individuals diagnosed with KOA; I (intervention)—cryotherapy associated or not with kinesiotherapy and/or compression; C (comparison)—simulated, placebo, no treatment or other physiotherapeutic intervention, except cryotherapy; O (outcome)—pain (primary), functionality, and muscle strength. S (study design)—randomized clinical trials.

Inclusion criteria: People diagnosed with KOA, men and women, without age restriction and with painful symptoms.

Exclusion criteria: Individuals undergoing surgical treatment for KOA, with cardiorespiratory and neurological medical restrictions, and conditions that prevented exercise were excluded.

The design of the studies included were randomized clinical trials, with no restrictions on period or language. If there was a need for other languages (except English, Spanish, Italian, and Portuguese) a translator would be used. Reports and case studies, systematic reviews, or any type of literature review, cohort studies, and studies that could not be read in full, even after contacting the author, were excluded.

### 2.3 Selection of Studies

Two independent reviewers, R1 and R2, selected the included articles in two phases. In the first phase (Phase 1), the two reviewers assessed the titles and abstracts according to the eligibility criteria; in the second phase (Phase 2), they examined the full texts. Finally, the studies included in this review were selected. In the event of disagreements between R1 and R2, a third reviewer (R3) took part in resolving the conflicts.

### 2.4 Data Collected

Data was collected on the characteristics of the studies (authors, year of publication, country), the sample (sample size, average age, and gender), intervention modality, evaluation and conclusion times. The primary outcome was pain and the secondary outcomes were functionality and muscle strength (Table 1).

**TABLE 1:** Summary characteristics of the randomized clinical trials included in this review (*n* = 05).

Eligible studies and country	Study design	Sample description	Intervention protocol	Evaluation periods	Measuring instruments and outcomes	Conclusion
Clarke et al., 1974 United Kingdom	Prospective, randomized	*N* = 28 EG: (*n* = 15) CG: (*n* = 13) Sex:: EG: M 27% CG: M 31% Age: EG: 64 CG: 63	EG: Cryotherapy CG: SWD not tuned	T0: Baseline T1: 3 weeks T2: 3 months	Pain intensity: (0–3)	Ice provided faster pain relief in the first 3 weeks for patients with knee osteoarthritis, but this difference was not maintained after 3 months, when all groups showed similar improvements
Silva et al., 2007 Brazil	Randomized prospective blind	*N* = 15 EG: (*n* = 06) CG: (*n* = 09) Sex: (F:19, M: 6) Age: 58–78	EG: Cryotherapy + exercise CG: Exercise	T0: Baseline T1: 5 weeks	Pain intensity: Borg—Functional quality: Lequesne (0–24) Muscle strength: sphygmomanometer	Pain improved significantly only in the experimental group, while function improved in all groups
Aciksoz, et al., 2017 Turkey		*N* = 64 EG: (*n* = 32) CG: (*n* = 32) Sex: EG: F 81.3% CG: F 78.1% Age: EG: 60.31 ± 8.37 CG: 63.50 ± 9.12	EG: Cryotherapy + exercise CG: Exercise	T0: Baseline T1: 2 weeks T2: 3 weeks	Pain intensity: VAS (0–10) WOMAC—pain (0–4) Disability: WOMAC‐disability (0–4)	The study concluded that the application of cold resulted in slight improvements in the pain and functional status of patients with primary osteoarthritis of the knee, although this was not statistically significant
Dantas et al., 2019 Brazil		*N* = 60 EG: (*n* = 30) CG: (*n* = 30) Sex: EG: (F: 15) CG: (F: 15) Age: EG: 60 ± 7 CG: 60 ± 7	EG: Cryotherapy + compression with elastic band CG: Sham cryotherapy	T0: Baseline T1: 6 days	Pain intensity: VAS (0–10) Physical function: WOMAC (0–96) KOOS (0–168)	Short‐term cryotherapy, combined with personalized therapeutic exercises, did not show a significant reduction in pain in patients with knee osteoarthritis and had uncertain effects on physical function
Mohammedsadiq & Rasool, 2023 Iraq	Prospective, randomized, double‐blind	*N* = 34 EG: (*n* = 18) CG: (*n* = 16) Sex: EG: (F:13 ± 72.22; M: 5 ± 27.78) CG: (F: 13 ± 81.25; M: 3 ± 18.75) Age: EG: 51.94 ± 5.84 CG: 51.38 ± 7.72	EG: HBE + cryotherapy CG: HBE	T0: Baseline T1: 2 months T2: follow up 3 months	Pain intensity: WOMAC (0–4) Physical function: WOMAC (0–4)	The study found that combining home‐based exercise with cryotherapy effectively improves function in knee osteoarthritis patients

Abbreviations: F, female; HBE, home‐based exercise; KOOS, Knee Injury and Osteoarthritis Outcome Score; M, male; SWD, short‐wave diathermy; WOMAC, Western Ontario and McMaster.

### 2.5 Individual Assessment of the Risk of Bias

The risk of bias assessment was carried out using the Cochrane tool, ROB 2, by two blinded reviewers R1 and R2. Disagreements were resolved by R3. All included studies were assessed in five domains: deviations from planned interventions, lack of outcome data, measurement of outcomes, selection of reported studies, and overall outcome of the bias analysis. Each domain had an overall result: low risk, some concern, or high risk.

### 2.6 Search Strategies

A comprehensive and sensitive search was carried out. The initial search was conducted using the keywords Cryotherapy and Knee Osteoarthritis in the PubMed database, with the medical metadata system Medical Subject Headings (MeSH) and also free terms. The following databases were consulted: PubMed, Web of Science, Embase, Cochrane, the Physiotherapy Evidence Database (PEDro), Scopus, and LILACS. Gray literature was also consulted via Google Scholar, LIVIVO, Open Gray, and the CAPES Catalog of Theses and Dissertations. The list of citations of the studies included in this review was also included.

### 2.7 Statistical Analysis

Statistical analysis was performed using RevMan 5.4.1 (The Cochrane Collaboration, Software Update, Oxford, UK) [21, 22]. Continuous results were expressed as mean differences with 95% confidence intervals (95% CI). A *p*‐value of < 0.05 was considered statistically significant. The Q^2^ and I^2^ statistical test values were calculated to test for heterogeneity between studies. An I^2^ values of 0%–40% might not be important; 30%–60% may represent moderate heterogeneity; 50%–90% may represent substantial heterogeneity; 75%–100% may represent considerable heterogeneity [21, 22]. A random effects model was adopted when statistically significant heterogeneity was identified. When necessary, transformations were used according to the Cochrane Handbook, Chapter 6 [23].

## 3 Results

### 3.1 Study Selection

**FIGURE 1:**
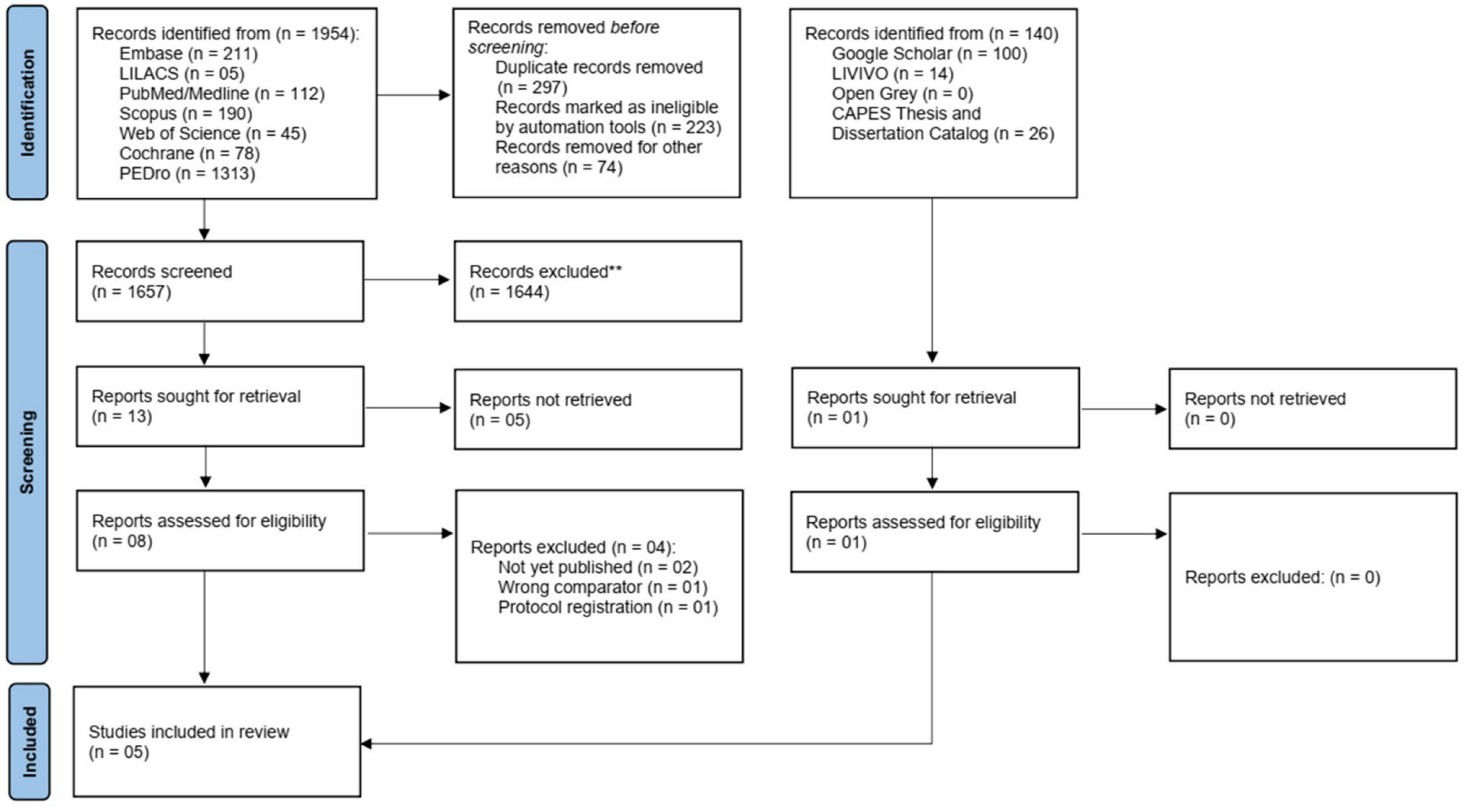
PRISMA 2020 flow diagram for new systematic reviews which included searches of databases, registers and other sources.

During the search, 2094 records were found, 1954 in the main databases and 140 in the gray literature (Appendix 1). The search was carried out in all databases on October 10, 2023 and updated on September 4, 2024. Of the 2094 records, 223 duplicate studies were automatically excluded from the indexed literature and two (02) from the gray literature. A further 74 were excluded from the indexed literature and four (04) from the gray literature manually. This left 1791 studies for Phase 1 (reading titles and abstracts). And 14 studies for Phase 2 (reading the full studies). This left 05 studies in this review (Figure 1).

### 3.2 Individual Study Results

Of the five studies included, two were conducted in Brazil [24, 25], one in the UK [26], one in Turkey [27] and one in Iraq [28]. All the studies included men and women, ranging in age from 43 to 78 years, with a total of 201 subjects. The individual results of this study are summarized in Table 1.

#### 3.2.1 Measuring Instruments

A variety of instruments were used to measure the outcomes of this study, some of them addressing both pain and functionality, and others specific to pain or functionality. Muscle strength was measured in only one study.

#### 3.2.2 Measuring Pain

The five studies analyzed pain with different instruments, but the interpretation of all of them was similar: the higher the score, the greater the intensity of pain.

Dantas et al. [25] and Aciksoz et al. [27] used the analog pain scale (VAS). Aciksoz et al. [27] also used the WOMAC questionnaire (0–4), as well as Mohammedsadiq & Rasool [28]. Clarke et al. [26] used a scale of Pain intensity (0–3). Silva et al. [24] a Borg pain intensity scale.

#### 3.2.3 Functionality/Disability

Aciksoz et al. [27] and Mohammedsadiq & Rasool [28] used the WOMAC disability (0–4). Dantas et al. [25] the physical function WOMAC (0–96) and Knee Injury and Osteoarthritis Outcome Score—KOOS (0–168). Likewise, Aciksoz et al. [27] used the Physical function WOMAC (0–96). Silva et al. [24] the Functional quality: Lequesne (0–24).

#### 3.2.4 Measuring Strength

Only the study by Silva et al. [24] assessed muscle strength using the modified sphygmomanometer test. In this method, the strength of the limbs was calculated using a pre‐established formula, where a higher percentage in the result indicates greater strength.

#### 3.2.5 Intervention Protocols

##### 3.2.5.1 Experiment vs. Control Groups

The study by Clarke et al. [26], used experimental and simulated groups, cryotherapy vs. non‐tuned short‐wave diathermy. The Silva et al. [24], Aciksoz et al. [27] and MohammedSadiq & Rasool [28], included cryotherapy vs. cryotherapy combined with an exercise program. Finally, Dantas et al. [25] cryotherapy + compression with elastic band vs. placebo with sandbags.

##### 3.2.5.2 Frequency and Duration of Treatment

There was no standardization regarding the duration and frequency of treatment in the clinical trials. The frequency varied between studies; Aciksoz et al. [27] performed treatment twice a day for 3 weeks, and up to three times a week over 8 weeks [28]. However, in relation to the length of time cryotherapy was applied, 4 studies [24, 25, 27, 28] applied ice for a period of 20 min per application, except Clarke et al. [26] who did not specify the period of application. More details can be seen in Table 2.

**TABLE 2:** Description of protocols and frequency of interventions.

Eligible studies	Intervention protocol
Clarke et al., 1974 [24, 26]	EG: Cryotherapy CG: Non‐tuned shortwave diathermy Both groups received the treatment 3 sessions a week for 3 weeks. It does not specify the exact time of ice application per session
Silva et al., 2007 [24]	EG: Cryotherapy + exercise CG: Exercise All groups received 10 sessions, twice a week, for 5 weeks. EG received cryotherapy for 20 min before kinesiotherapy
Aciksoz et al., 2017 [27]	EG: Cryotherapy + exercise CG: Exercise EG/CG: NSAIDs, paracetamol‐type drugs, topical creams Cold package for 20 min 2 times a day, in the morning and in the evening, for 3 weeks
Dantas et al., 2019 [25]	EG: Cryotherapy + compression with elastic band CG: Placebo with sandbags In both groups, four sessions were carried out over four consecutive days for 20 min on the most affected knee
MohammedSadiq & Rasool, 2023 [28]	EG: Cryotherapy + home exercises + education about the risk factors of KOA CG: Home exercises + education about the risk factors of KOA 8 weeks/3 sessions/week on non‐consecutive days/totaling 24 sessions Cryotherapy 20 min

### 3.3 Analysis of the Risk of Bias

**FIGURE 2:**
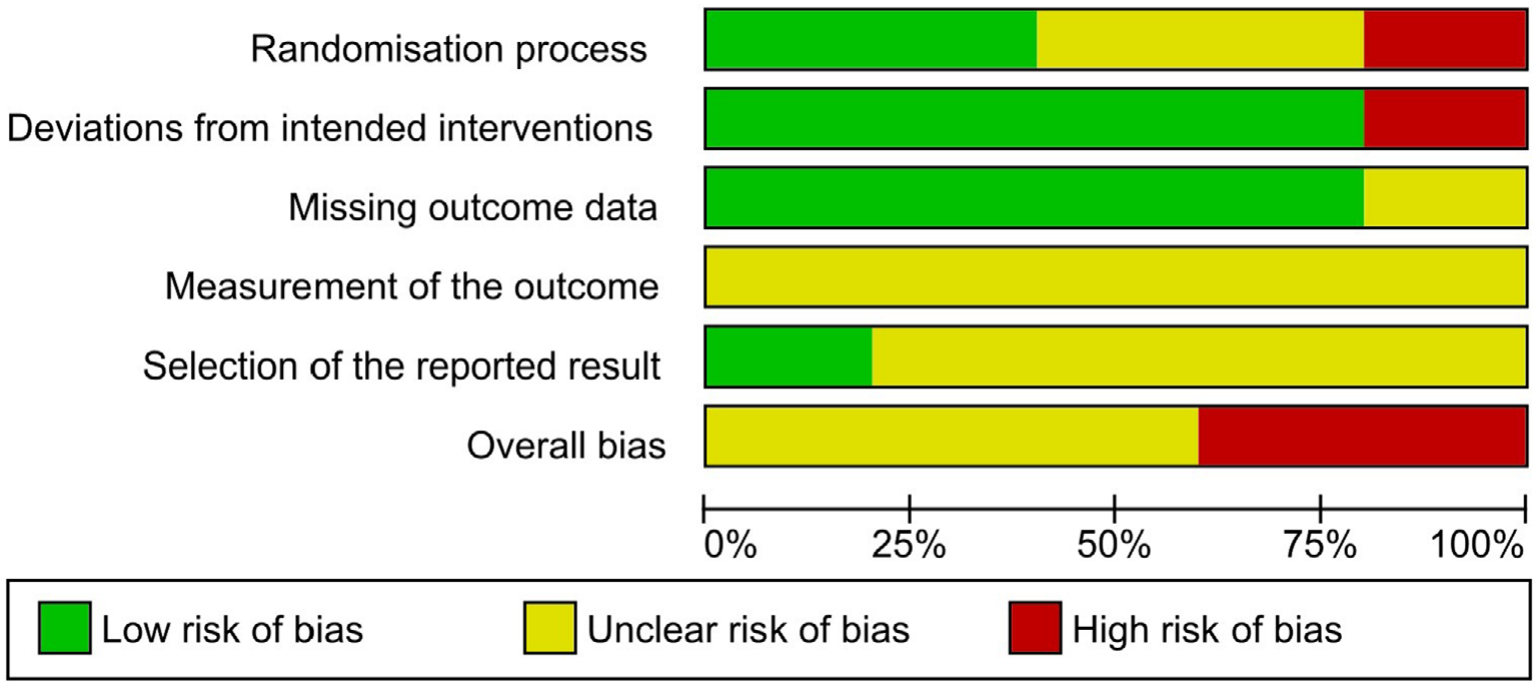
Risk of bias graph: Review authors' judgments about each risk of bias item presented percentages across all included studies.

Figure 2 presents the risk of bias, highlighting that the randomization process and the collection of outcome data were largely conducted with a low risk of bias, although there were a few cases of some concerns and high risk. Deviations in the interventions and the measurement of outcomes presented some concerns, suggesting possible inconsistencies or lack of transparency. The selection of the results reported also raised some concerns, with a considerable risk of some concerns and some high risk, which may indicate the possibility of a selective choice of the data presented.

**FIGURE 3:**
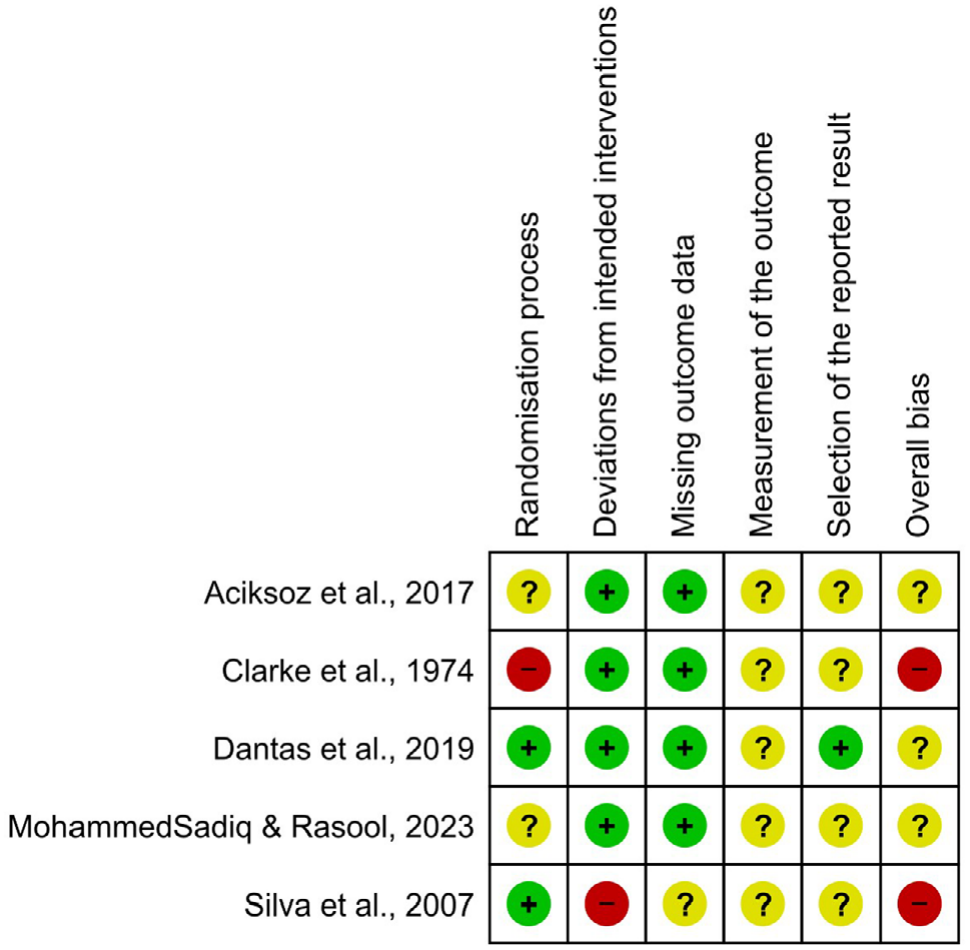
Risk of bias summary: Review authors' judgments about each risk of bias item for each included study.

Analysis of the five studies in relation to the different domains of bias reveals significant variations. With regard to the randomization process, the studies by Dantas et al. [25] and Aciksoz et al. [27] have a low risk of bias, while studies of Clarke et al. [26] and Silva et al. [24] have a high risk, indicating possible methodological problems. With regard to deviations from the intended interventions, all the studies except Aciksoz et al. [27] show a low risk of bias, suggesting that the interventions were applied according to protocol. Most studies also show a low risk of bias in terms of missing outcome data, except for Silva et al. [24], who raised some concerns. When measuring outcomes, Dantas et al. [25] and Clarke et al. [26] showed a low risk, while the other studies show some concerns, raising questions about the accuracy of the results. Regarding the selection of reported results, all the studies presented some concerns or a high risk, which may indicate problems with transparency or consistency in the presentation of results (Figure 3).

### 3.4 Metanalysis

**FIGURE 4:**
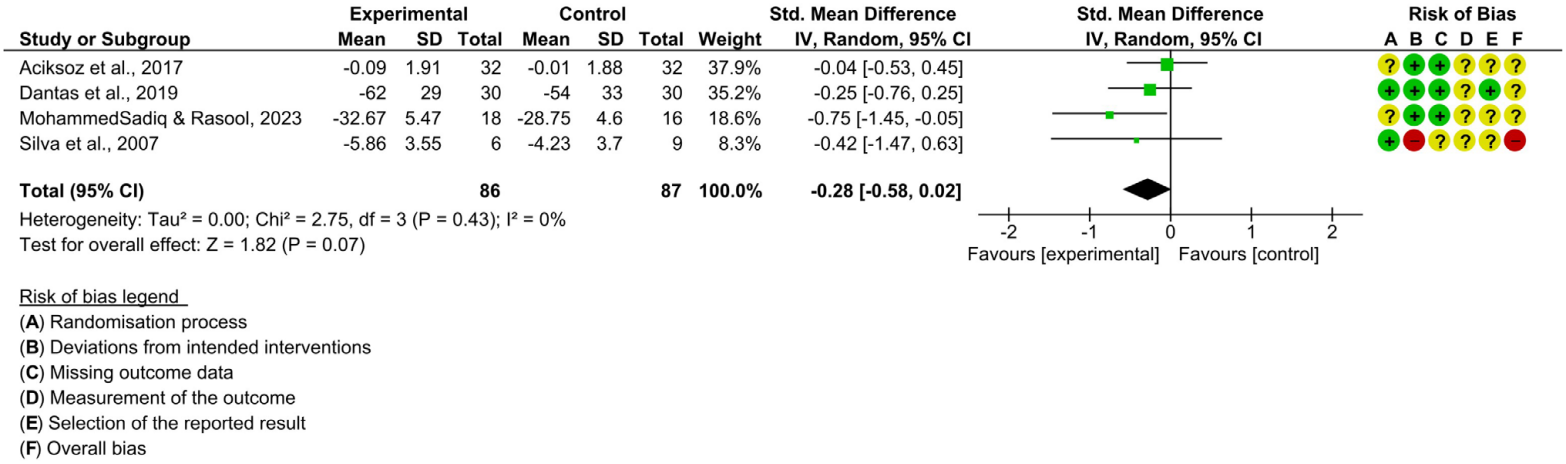
Forest plot comparing cryotherapy vs. control—pain intensity outcome.

The result of the meta‐analysis of the pain intensity outcome included the five clinical trials [24, 25, 26, 27, 28] with 201 individuals. The pooled effect estimate between the studies for the pain intensity outcome (SMD = −0.57; 95% (95%) CI; [−0.97, −0.18]; *p* = 0.004; I^2^ = 42%) Figure 4. Thus, the cryotherapy intervention was significant in relation to the control group for the pain intensity outcome.

**FIGURE 5:**
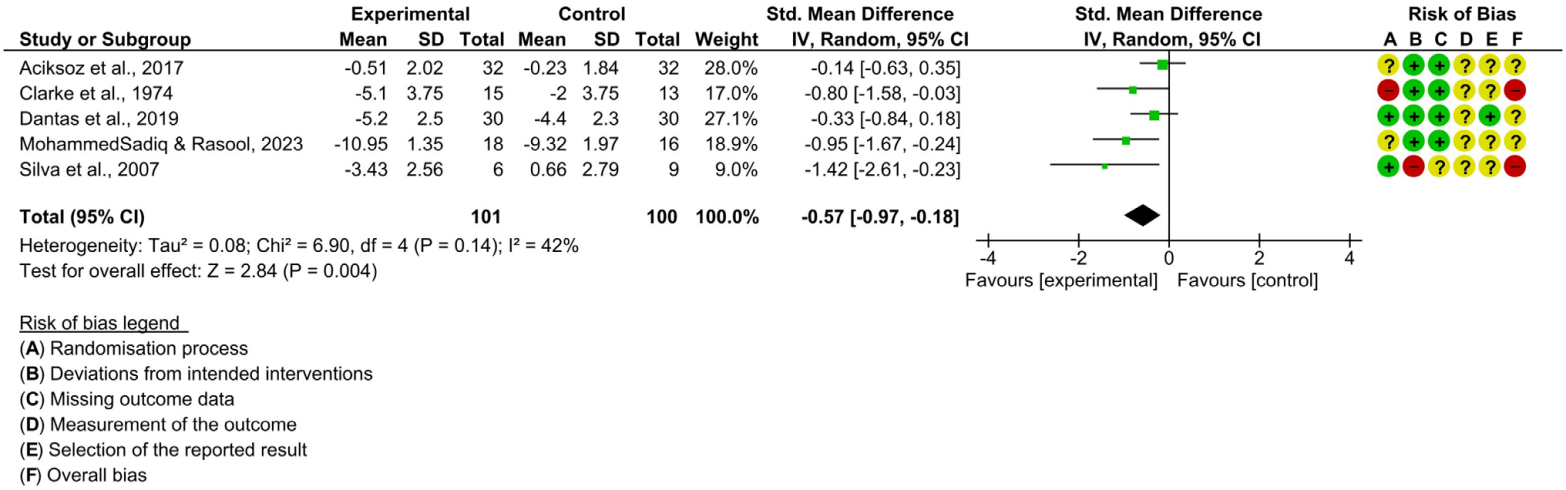
Forest plot of comparison between cryotherapy + exercise vs. control—functionality outcome.

As for functionality, the results indicate the combination of effect estimates between four clinical trials [24, 25, 27, 28], with 173 individuals, on the functionality outcome (SMD = −0.28; 95% (95%) CI; [−0.58, 0.02]; *p* = 0.07; I^2^ = 0%). Suggesting a slight trend in favor of the experimental groups, although the overall result was not statistically significant (*p* = 0.07). And the analysis suggests homogeneity in the effects observed between the studies I^2^ = 0%, suggests homogeneity between the studies Figure 5.

## 4 Discussion

This study aimed to update and expand the literature on the effects of cryotherapy on pain, muscle strength and function in individuals with knee OA. It has been observed that cryotherapy is an appropriate method, especially when combined with others such as kinesiotherapy. Searches were carried out in seven indexed databases in addition to the gray literature, as this is often neglected and should always be included in order to generate a balanced view of the evidence [29]. Even with this expansion, only five studies were included, in three of which cryotherapy was associated with physical exercise [24, 27, 28] and two were cryotherapy alone [25, 26].

Cryotherapy is a therapy that aims to reduce body temperature and thus reduce body metabolism, nerve conduction, blood flow and is consequently important for producing analgesia and reducing the inflammatory process [13, 16, 17, 18]. For osteoarthritis of the knee, Zhang et al. [19] point out that the issue is controversial, with studies indicating its use while others contraindicate it. However, despite controversy, there may be a reduction in muscle strength and proprioception, which is obviously reversible as tissue temperature returns [30, 31].

Of the five primary clinical trials included, it is worth noting that none had a low overall risk of bias. The tool used to analyze the risk of bias was ROB 2, specific for randomized clinical trials, subdivided into five topics. Two studies presented high risks of bias, while the other three had some concerns. This points to the need for greater care in the design of future studies, with a view to improving the methodological quality of the studies.

Aiming for a more in‐depth analysis of the subject, two meta‐analyses were carried out, the first of which showed that cryotherapy, whether or not associated with physical exercise, had a significant effect on reducing pain intensity when compared to the control group. This result, although significant, should be interpreted with caution due to the moderate heterogeneity observed between the studies (I^2^ = 42%). This suggests that although there is a general benefit of cryotherapy in reducing pain, the variability in the interventions and populations studied may have influenced the results.

The second meta‐analysis was in relation to functionality; cryotherapy did not show a significant effect when analyzed with other interventions such as exercise. The SMD value (−0.28) was close to zero, indicating a minimal difference between the experimental and control groups. Furthermore, the homogeneity observed (I^2^ = 0%) suggests that the results were consistent between the studies, reinforcing the conclusion that cryotherapy alone may not be enough to significantly improve functionality in patients with KOA. It is worth noting that the *p*‐value was 0.07, i.e., close to a statistical difference, which may indicate that the small number of studies included was the cause of the lack of statistical significance, and this result may have some clinical relevance, since on average, three studies showed results favorable to the experiment.

As for muscle strength, only one of the studies reviewed addressed this outcome, using the modified sphygmomanometer test. The results did not indicate a significant improvement in muscle strength associated with the application of ice, which limits the ability to generalize the effects of cryotherapy on this aspect of KOA.

Given these results, it can be inferred that cryotherapy may be a beneficial intervention in reducing pain in patients with KOA, but its effectiveness in terms of improving functionality and muscle strength remains uncertain. The lack of standardization in intervention protocols and the variability in the measurement instruments used are factors that contribute to this uncertainty. Another limitation of this study was the inclusion of few studies in the meta‐analysis, which weakens its findings. Therefore, future studies with greater methodological rigor and greater homogeneity in treatment protocols are needed to establish more definitively the role of cryotherapy in the management of knee osteoarthritis. However, it is recommended that a meta‐analysis can be conducted whenever there are at least two clinically similar studies, even if they are few in number, as long as there is minimal methodological homogeneity and comparability between the outcomes [23]. The use of appropriate models (random effects), the analysis of heterogeneity, and the consistency between the measuring instruments used ensured the reliability of the findings, which were interpreted with due caution.

## 5 Conclusion

These findings suggest that cryotherapy can be considered as part of a broader treatment plan for OAJ, especially when combined with other therapeutic modalities such as kinesiotherapy. However, its effectiveness as a stand‐alone treatment still needs more robust evidence.

## Author Contributions

All listed authors should have contributed to the manuscript substantially and have agreed to the final submitted version. R.F.D., S.L.S., S.P.M., L.J.F.F., M.R.B., G.R.F.B.: concept or design of the article; R.F.D., S.L.S., S.P.M.: data acquisition and analysis; L.J.F.F., M.R.B., G.R.F.B.: interpretation of data for the article; R.F.D., S.L.S., S.P.M.: writing the manuscript; L.J.F.F., M.R.B., and G.R.F.B.: critical revision of the manuscript; R.F.D., S.L.S., S.P.M., L.J.F.F., M.R.B., G.R.F.B.: approval of the final version.

## Ethics Statement

The proposed systematic review collects and analyzes secondary data that is associated with individuals. Each primary study included should have been approved by an ethics committee to carry out the clinical trial.

## Consent

The authors have nothing to report.

## Conflicts of Interest

The authors declare no conflicts of interest.

## Data Availability

The data that support the findings of this study are available on request from the corresponding author. The data are not publicly available due to privacy or ethical restrictions.

## Appendix 1 Search Strategy for the Databases

**Table:**

Databases	September/04/24
PubMed	Search: ((“Osteoarthritis, Knee”[Mesh] OR “Osteoarthritis, Knee”) OR (“Osteoarthritis, Knee”[Title/Abstract]) OR “Knee Osteoarthritides” OR “Knee Osteoarthritis” OR “Osteoarthritis of Knee” OR “Osteoarthritis of the Knee”) AND ((Cryotherapy[Mesh] OR Cryotherapy) OR (Cryotherapy[Title/Abstract]) OR Cryotherapies OR “Cold Therapy” OR “Cold Therapies” OR Cryostimulation OR “Cold Temperature”[Mesh] OR “Cold Temperature”))
Web of Science	(“Osteoarthritis, Knee” OR “Knee Osteoarthritides” OR “Knee Osteoarthritis” OR “Osteoarthritis of Knee” OR “Osteoarthritis of the Knee”) (Tópico) and (Cryotherapy OR cryotherapie OR “Cold Therapy” OR “Cold Therapies” OR “Cryostimulation” OR “Cold Temperature”) (Tópico)
Scopus	TITLE‐ABS‐KEY (“Osteoarthritis, Knee” OR “Knee Osteoarthritides” OR “Knee Osteoarthritis” OR “Osteoarthritis of Knee” OR “Osteoarthritis of the Knee”) AND TITLE‐ABS‐KEY (cryotherapy OR cryotherapies OR “Cold Therapy” OR “Cold Therapies” OR “Cryostimulation” OR “Cold Temperature”
Embase	(“osteoarthritis, knee” OR “knee osteoarthritides” OR “knee osteoarthritis” OR “osteoarthritis of knee” OR “osteoarthritis of the knee”) AND (“cryotherapy”/exp. OR cryotherapy OR cryotherapies OR “cold therapy”/exp. OR “cold therapy” OR “cold therapies” OR “cryostimulation” OR “cold temperature”/exp. OR “cold temperature”)
Lilacs	((“Osteoarthritis, Knee” OR “Knee Osteoarthritides” OR “Knee Osteoarthritis” OR “Osteoarthritis of Knee” OR “Osteoarthritis of the Knee” OR “Osteoartritis de la Rodilla” OR “Osteoartrite de joelho” OR “Artrosis de Rodilla” OR “Artrosis de la Rodilla” OR gonartrosis OR “Osteoartritis de Rodilla” OR “Artrose de joelho” OR “Artrose do joelho” OR gonartrose OR “Osteoartrite de joelho”)) AND ((cryotherapy OR cryotherapies OR “Cold Therapy” OR “Cold Therapies” OR cryostimulation OR “Cold Temperature” OR crioterapia)) AND (db:(“LILACS”))
Cochrane	(“Osteoarthritis, Knee” OR “Knee Osteoarthritides” OR “Knee Osteoarthritis” OR “Osteoarthritis of Knee” OR “Osteoarthritis of the Knee”) in Title Abstract Keyword AND (Cryotherapy OR Cryotherapies OR “Cold Therapy” OR “Cold Therapies” OR “Cryostimulation” OR “Cold Temperature”) in Title Abstract Keyword ‐ (Word variations have been searched)
PeDro	(Electrotherapies, heat, cold) AND (lower leg or knee)
Google scholar	(“Osteoarthritis, Knee” OR “Knee Osteoarthritides” OR “Knee Osteoarthritis” OR “Osteoarthritis of Knee” OR “Osteoarthritis of the Knee” OR “Osteoartritis de la Rodilla” OR “Osteoartrite de joelho” OR “Artrosis de Rodilla” OR “Artrosis de la Rodilla” OR Gonartrosis OR “Osteoartritis de Rodilla” OR “Artrose de joelho” OR “Artrose do joelho” OR Gonartrose OR “Osteoartrite de joelho”) AND (Cryotherapy OR Cryotherapies OR “Cold Therapy” OR “Cold Therapies” OR Cryostimulation OR “Cold Temperature” OR Crioterapia)
Open Gray	(Knee Osteoarthritides) AND (Cryotherapy)
LIVIVO	(“Osteoarthritis, Knee” OR “Knee Osteoarthritides” OR “Knee Osteoarthritis” OR “Osteoarthritis of Knee” OR “Osteoarthritis of the Knee”) AND (Cryotherapy OR Cryotherapies OR “Cold Therapy” OR “Cold Therapies” OR “Cryostimulation” OR “Cold Temperature”)
CAPES Thesis and Dissertation Catalog	(Osteoartrite de joelho) AND (Crioterapia)
